# Differential regulation of nimodipine-sensitive and -insensitive Ca^2+^ influx by the Na^+^/Ca^2+^ exchanger and mitochondria in the rat suprachiasmatic nucleus neurons

**DOI:** 10.1186/s12929-018-0447-z

**Published:** 2018-05-22

**Authors:** Pi-Cheng Cheng, Yi-Chi Wang, Ya-Shuan Chen, Ruo-Ciao Cheng, Jyh-Jeen Yang, Rong-Chi Huang

**Affiliations:** 1 Department of Physiology and Pharmacology, College of Medicine, Chang Gung University, 259 Wen-Hwa 1st Road, Kwei-San, Tao-Yuan, 33302 Taiwan; 2 Healthy Aging Research Center, Chang Gung University, Tao-Yuan, 33302 Taiwan; 3 Neuroscience Research Center, Chang Gung Memorial Hospital, Linkou Medical Center, Tao-Yuan, 33305 Taiwan

**Keywords:** Action potential, Ca^2+^ channels, Ca^2+^ imaging, Na^+^/Ca^2+^ exchanger, Mitochondria, Suprachiasmatic nucleus

## Abstract

**Background:**

Transmembrane Ca^2+^ influx is critical for molecular rhythmicity, metabolic activity, and neuropeptide release in the central clock of the suprachiasmatic nucleus (SCN). We previously reported that both the Na^+^/Ca^2+^ exchanger (NCX) and mitochondria play a role in regulating intracellular Ca^2+^ homeostasis in the rat SCN neurons. Here we present evidence to show differential regulation by NCX and mitochondria of nimodipine-sensitive and -insensitive Ca^2+^ influx.

**Methods:**

Ratiometric Ca^2+^ imaging was used to measure change in [Ca^2+^]_i_ and patch clamp recordings to study spontaneous firing, membrane potential, and voltage-dependent Ca^2+^ channels in neurons from reduced SCN slice preparations. Immunofluorescent staining was used to determine the distribution pattern of CaV1.2 and CaV1.3 and their colocalization with NCX1.

**Results:**

Ratiometric Ca^2+^ imaging indicates that nimodipine (2 μM) blocked most of 20 (mM) K^+^-induced, but less so of 50 K^+^-induced, Ca^2+^ rise. The nimodipine-sensitive 50 K^+^-induced Ca^2+^ transient rose more rapidly but decayed similarly with the nimodipine-insensitive component, suggesting both components were extruded by NCX. Immunofluorescent stains showed the expression of both CaV1.2 and CaV1.3 and their colocalization with NCX1, whereas functional studies suggest that CaV1.2 mediated most of the nimodipine-sensitive Ca^2+^ rise but had insignificant effect on spontaneous firing. After normalization relative to the Ca^2+^-free solution, nimodipine reduced ~ 65% of basal Ca^2+^ influx, and TTX lowered it by ~ 35%, leaving ~ 25% basal Ca^2+^ influx in the combined presence of TTX and nimodipine. With the mitochondrial uncoupler carbonyl cyanide-p-trifluoromethoxyphenylhydrazone (FCCP) to inhibit mitochondrial Ca^2+^ uptake, 20 K^+^-induced Ca^2+^ transients became larger and slower, both in the absence and presence of nimodipine. FCCP markedly enhanced nimodipine-insensitive, but not nimodipine-sensitive, Ca^2+^ transients, suggesting that mitochondria preferentially buffer nimodipine-insensitive Ca^2+^ influx. Results from using CaV2 channel blockers further indicate that FCCP enhanced Ca^2+^ transients mediated by N-, P/Q-, and the blocker cocktail-insensitive Ca^2+^ channels.

**Conclusions:**

The differential regulation of transmembrane Ca^2+^ influx by NCX and mitochondria suggests that Ca^2+^ entry via different sources may be regulated differently to play different roles in SCN physiology.

## Background

The hypothalamic suprachiasmatic nucleus (SCN) is the central clock controlling circadian rhythms in mammals [1]. The SCN neurons exhibit a circadian rhythm in spontaneous firing rate [2–5], [Ca^2+^]_i_ [6–8], and metabolic activity such as 2-deoxyglucose uptake [9, 10], cytochrome oxidase activity [11], and Na^+^/K^+^-ATPase pumping activity [12]. Importantly, the daytime increase in spontaneous firing and [Ca^2+^]_i_ is required for the activation of Ca^2+^/cAMP-response element (CRE) to sustain molecular rhythmicity [13] (for review, see ref. [14]), the increase in 2-deoxglucose uptake [9, 15], and the increased in vivo release of the three major neuropeptides, arginine vasopressin (AVP), vasoactive intestinal peptide (VIP), and gastrin-releasing peptide (GRP) in the SCN [16, 17]. For example, the removal of external Ca^2+^ and/or addition of combined blockers for voltage-dependent Ca^2+^ channels abolishes the molecular rhythmicity [18] and reduces the resting release of AVP, VIP, and GRP [16, 17]. Blocking Na^+^-dependent action potentials with TTX reduces oscillation in [Ca^2+^]_i_, CRE activation, and clock gene expression [7, 13, 19, 20].

Nevertheless, intracellular Ca^2+^ homeostasis relies on the functional interaction of various Ca^2+^ handling systems, such as those involved in mediating Ca^2+^ entry (voltage- and receptor-operated Ca^2+^ channels), extrusion (plasmalemmal Na^+^/Ca^2+^ exchanger (NCX) and Ca^2+^-ATPase (PMCA)), and buffering (Ca^2+^ binding proteins, endoplasmic reticulum, and mitochondria). In a previous study, we demonstrated an important role of NCX and mitochondria in clearing depolarization-induced Ca^2+^ rise, with NCX mediating fast Ca^2+^ decay following high K^+^-induced Ca^2+^ transients [21]. We found that the blockade of NCX with Na^+^-free solution slowed threefold the fast decay kinetics of Ca^2+^ transients. In contrast, blockade of PMCA, sarco(endo)plasmic reticulum Ca^2+^-ATPase (SERCA), ryanodine receptors, or IP3 receptors had little effect on the decay kinetics of Ca^2+^ transients. Nevertheless, drugs used to block SERCA (1 μM thapsigargin), ryanodine receptors (10 μM dantrolene), and IP3 receptors (100 μM 2-aminoethoxydiphenyl borate) also lowered basal [Ca^2+^]_i_ and reduced the peak amplitude of Ca^2+^ transients to various extents. As these drugs are known to block L-type Ca^2+^ channels [22, 23], the result suggested a contribution of L-type Ca^2+^ channels to basal [Ca^2+^]_i_ and depolarization-induced Ca^2+^ rise.

The neuronal nimodipine-sensitive L-type Ca^2+^ channels, CaV1.2 and CaV1.3, play important roles in synaptic neurotransmission, neuronal firing, and regulation of gene expression [24]. In the rat SCN neurons, whereas L-type channels only occupy a small proportion of total voltage-activated Ca^2+^ currents [25–28], their activation at more negative potentials contribute significantly to depolarization-dependent Ca^2+^ rise [27]. Indeed, L-type Ca^2+^ channels contribute to basal levels of Ca^2+^ in the rat SCN neurons [21, 29] and their blockade with nimodipine reduces the oscillation amplitude of *Per2* clock gene [19]. Nevertheless, the contribution of L-type channels to spontaneous firing remains unsettled, with 2 or 10 μM nimodipine having insignificant effect on the rat SCN neurons in dissociation [27] and in slices [25], but reducing the firing rate in both mouse and rat SCN neurons in slices [28, 30].

While both CaV1.2 and CaV1.3 are found in the mouse SCN [30, 31], it is not known whether both channel types are expressed in the rat SCN neurons. It is also not entirely clear how they contribute to transmembrane Ca^2+^ influx and spontaneous firing in the rat SCN neurons. In this study, we determined the TTX- and/or nimodipine-sensitive proportions of the basal Ca^2+^ influx in the rat SCN neurons. We examined the immunostaining of CaV1.2 and CaV1.3 in the rat SCN and determined the concentration-dependent effects of nimodipine on transmembrane Ca^2+^ influx and neuronal firing. We also tested the idea that mitochondria might differentially buffer nimodipine-sensitive and -insensitive Ca^2+^ influx. The experiments were performed during the day when spontaneous firing rate, [Ca^2+^]_i_, and metabolic activity are higher compared to the night. The immunostaining results showed the expression of CaV1.2 and CaV1.3 and their colocalization with NCX1, whereas functional data revealed a major contribution of CaV1.2 to transmembrane Ca^2+^ influx but not to spontaneous firing. Interestingly, there remained a portion of basal Ca^2+^ influx that is insensitive to the combined presence of TTX and nimodipine. Our results indicate that while NCX extrudes depolarization-induced Ca^2+^ influx via both nimodipine-sensitive and -insensitive Ca^2+^ channels, mitochondria regulate [Ca^2+^]_i_ by preferentially buffering nimodipine-insensitive Ca^2+^ influx.

## Methods

### Hypothalamic brain slices and reduced SCN preparations

All experiments were carried out according to precedures approved by the Institutional Animal Care and Use Committee of Chang Gung University. Sprague-Dawley rats (18–24 days old) were kept in a temperature-controlled room under a 12:12 light:dark cycle (light on 0700–1900 h). Lights-on was designated Zeitgeber time (ZT) 0. All experiments were performed at day and the animal was killed at ZT 2. An animal of either sex was carefully restrained by hand to reduce stress and killed by decapitation using a small rodent guillotine without anaesthesia, and the brain was put in an ice-cold artificial cerebrospinal fluid (ACSF) prebubbled with 95% O_2_-5% CO_2_. The ACSF contained (in mM): 125 NaCl, 3.5 KCl, 2 CaCl_2_, 1.5 MgCl_2_, 26 NaHCO_3_, 1.2 NaH_2_PO_4_, 10 glucose. A coronal slice (200–300 μm) containing the SCN and the optic chiasm was cut with a DSK microslicer DTK-1000 (Ted Pella, Redding, CA, USA), and was then incubated at room temperature (22–25 °C) in the incubation solution, which contained (in mM): 140 NaCl, 3.5 KCl, 2 CaCl_2_, 1.5 MgCl_2_, 10 glucose, 10 HEPES, pH 7.4, bubbled with 100% O_2_.

For electrical recordings and fluorescent Ca^2+^ imaging, a reduced SCN preparation was obtained by excising a small piece of tissue (circa one-ninth the size of SCN) from the medial SCN using a fine needle (Cat no. 26002-10, Fine Science Tools, Foster City, CA, USA), followed by further trimming down to 4–10 smaller pieces with a short strip of razor blade. The reduced preparation (containing tens of cells, see Fig. 1 of ref. [21]) was then transferred to a coverslip precoated with poly-D-lysine (Sigma-Aldrich, St Louis, MO, USA) in a recording chamber for recording. The SCN neurons of the reduced preparation could be identified visually with an inverted microscope (Olympus IX70 and IX71, Japan). The preparation thus obtained allows rapid application of drugs [32] and has been used for fluorescent Na^+^ and Ca^2+^ imaging [21, 33] and to demonstrate diurnal rhythms in both spontaneous firing and Na/K pump activity [12].

**Fig. 1:**
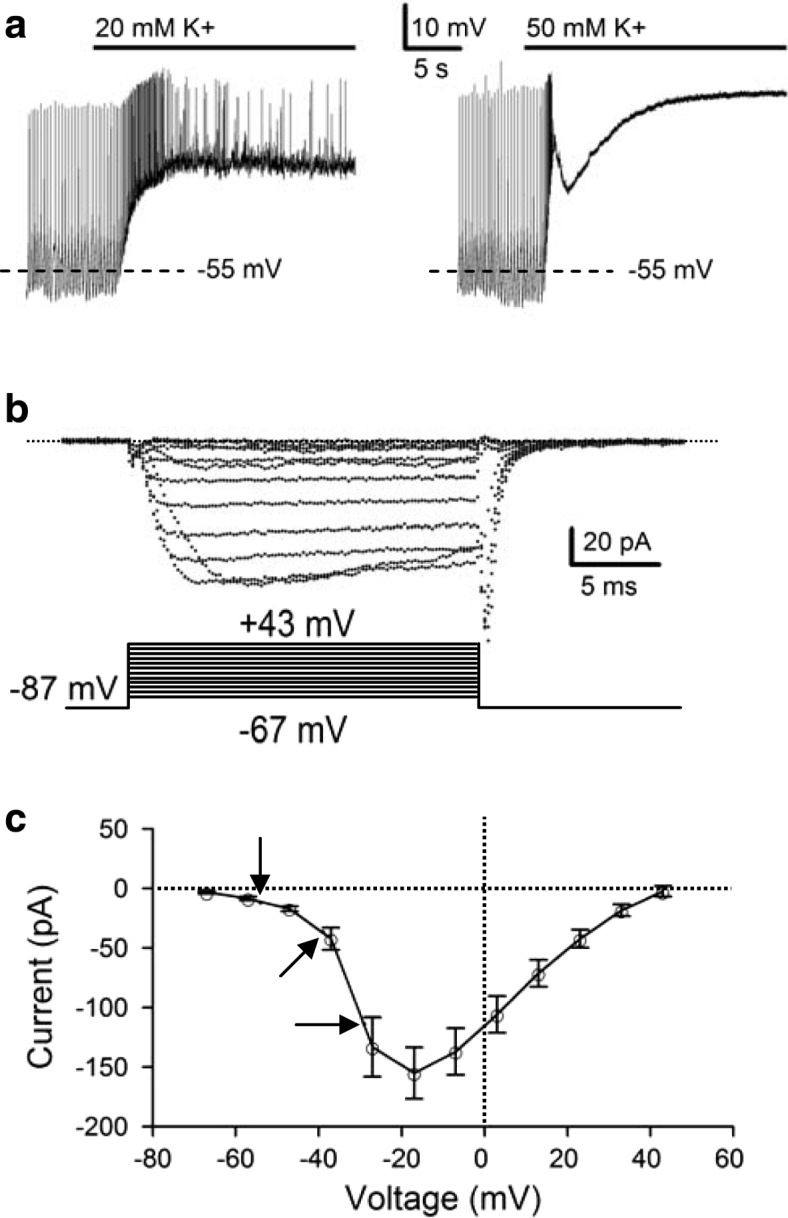
High K^+^-induced membrane depolarizations and voltage dependence of Ca^**2**+^ channels. **a** Voltage responses to 20 (*left* panel) and 50 (*right* panel) mM K^+^ solution from a representative SCN neuron recorded with the perforated patch technique. **b** Family of calcium currents activated from a holding potential of − 87 mV recorded with the whole-cell technique. Lower panel: voltage-clamp protocol. Horizontal dotted line was the zero current level. **c** Summary of experiments showing the *I-V* relation. The Ca^**2**+^ current amplitude was measured by taking the average near the end of 20-ms step. Each data point represents the mean ± SEM of 9 cells. Arrows marked the average membrane potentials at rest (downward arrow) and in response to 20 mM (slant arrow) and 50 mM (horizontal arrow) K^+^

### Electrical recordings

The reduced SCN preparation was perfused with bath solution containing (in mM): 140 NaCl, 3.5 KCl, 2 CaCl_2_, 1.5 MgCl_2_, 10 glucose, 10 HEPES, pH adjusted to 7.4 with NaOH. All recordings were made with Axopatch 200B amplifier (Axon Instruments, Foster City, CA, USA) at room temperature (22–25 °C). The spontaneous firing rate was recorded in the cell-attached configuration. The patch electrode was filled with the bath solution or with the patch solution containing (in mM): 20 NaCl, 1 CaCl_2_, 2 MgCl_2_, 110 K-gluconate, 11 EGTA, 10 HEPES, 3 Na-ATP, 0.3 Na-GTP, pH adjusted to 7.3 with KOH. The spike counts, in 6-s epochs, always began only after stable recordings were made. At least one or 2 min of spontaneous firing rate were counted before the application of drugs. Membrane potentials were recorded in the perforated patch current-clamp configuration. For perforated patch recordings, the patch pipette also included nystatin (Sigma-Aldrich, St Louis, MO, USA) at a final concentration of 250 μg/ml prepared from a stock solution (25 mg/ml DMSO). Pipette resistance was 4–6 MΩ. The measured liquid junction potential was − 12 mV and was corrected for in the presentation of data. Membrane currents were recorded in the whole-cell voltage-clamp configuration. For recording calcium currents in the whole-cell mode, the bath solution contained (in mM): 150 TEACl, 2 CaCl_2_, 10 HEPES, pH adjusted to 7.4 with TEAOH. The patch electrode contained (in mM): 90 *N*-methyl-D-glucamine (NMG), 45 NMGCl, 7.5 EGTA, 9 HEPES, 1.8 MgCl_2_, 4 Mg-ATP, 0.3 GTP, 14 Creatinine phosphate, pH adjusted to 7.4 with H_3_PO_4_. Pipette resistance was 5–8 MΩ. The measured liquid junction potential was − 7 mV and was corrected for in the measurements of voltages. The signal was low-pass filtered at 1–5 KHz (2–5 KHz for recording Ca^2+^ currents) and digitized on-line at 2–10 KHz (5–20 KHz for Ca^2+^ current recordings) via a 12-bit A/D digitising board (DT2821F-DI, Data Translation, Marboro, MA, USA) with a custom-made program written in the C Language.

### Ca^2+^ imaging

Fluorescent Ca^2+^ imaging was performed by pre-loading the SCN neurons with the Ca^2+^-sensitive fluorescent indicator Fura-2 acetoxymethyl ester (Fura-2 AM) [34]. The reduced SCN preparation was incubated in 10 μM Fura-2 AM (Molecular Probe, Invitrogen, Carlsbad, CA, USA) in 50 μl of bath solution in the dark for 60 min at 37 °C. Incubation was terminated by washing with 6 ml of bath solution and at least 60 min was allowed for de-esterification of the dye. All imaging experiments were performed at room temperature (22–25 °C). For the experiments, the reduced SCN preparation was gently pressed on the edge against the coverslip to allow adherence of the tissue to the surface. Fluorescence signals were imaged using a charge-coupled device camera (Olympus XM10, Japan) attached to an inverted microscope (Olympus IX71, Japan) and recorded with Xcellence imaging software integrated with the CellIR MT20 illumination system (Olympus Biosystems, Planegg, Germany). The system used a 150 W xenon arc burner as the light source to illuminate the loaded cells. The excitation wavelengths were 340 (± 12) nm and 380 (± 14) nm, and emitted fluorescence was collected at 510 nm. Pairs of 340/380 nm images were sampled at 0.5 Hz. Ca^2+^ levels in regions of interest (ROI) over the soma were spatially averaged and presented by fluorescence ratios (F340/F380) after background subtraction (see Fig. 1 of ref. [21]). Data were analyzed and plotted with custom-made programs written in Visual Basic 6.0 and the commercial software GraphPad PRISM (GraphPad Software, San Diego, CA, USA). All experiments were repeated at least five times. Data were given as means ± SEM and analyzed with Student’s *t*-test or paired *t*-test or ANOVA, followed by Tukey’s test for comparison of selected pairs.

### Drugs

Stock solutions of nimodipine (20 mM in DMSO), TTX (0.3 mM in acetic acid), and carbonyl cyanide-p-trifluoromethoxyphenylhydrazone (FCCP; 1 mM in DMSO) were stored at –20 °C, and were diluted at least 1000 times to reach desired final concentrations. These chemicals were purchased from Tocris Cookson (Ellisville, MO, USA). The CaV2 channel blockers SNX-482, ω-agatoxin IVA, and ω-conotoxin GVIA were purchased from Alomone Labs (Jerusalem, Israel) and were added directly to the bath solution. K^+^-free solution was prepared without extracellular K^+^, Ca^2+^-free solution was prepared with omission of extracellular Ca^2+^ and the addition of 1 mM EGTA, and high (20, 35, and 50 mM) K^+^ solutions were prepared with equal molar substitution of K^+^ for Na^+^.

### Immunofluorescence

Sprague-Dawley rats (23–25 days old) were deeply anesthetized with Zoletil (40 mg/kg, i.p.; Virbac Laboratories, Carros, France) and fixed by transcardial perfusion with PBS and then with 4% paraformaldehyde (500 ml/animal). Brains were removed and post-fixed overnight (more than 16 h) in 4% paraformaldehyde, followed by dehydration with 30% sucrose in PBS for another 24 h. Twenty-micrometer-thick coronal sections through the hypothalamus region containing the SCN were cut on a cryostat (–20 °C), collected in antifreeze solution, and stored in − 20 °C freezer until further processing.

For immunofluorescence staining, sections (20 μm) were washed for 20–30 min in PBS and then incubated overnight at 4 °C in PBS containing 2% serum, 0.3% Triton X-100, and primary antibodies against NCX1 (mouse anti-NCX1, against epitope between amino acid 371 and 525 on intracellular side of plasma membrane; 1:100; AB2869; Abcam, MA, USA) [21, 35]; CaV1.2 (rabbit anti-CaV1.2; 1:100; C-1603; Sigma-Aldrich, St Louis, MO, USA) [36]; CaV1.3 (rabbit anti-CaV1.3; 1:100; C-1728; Sigma-Aldrich, St Louis, MO, USA) [36]. Sections were then treated with Alexa Fluor secondary antibodies 488 or 568 (diluted 1:200; Molecular Probes, Eugene, OR, USA) and Hoechst 33,342 (B-2261; Sigma, St. Louis, MO, USA) for 1 h at room temperature. After rinse in PBS, sections were coverslipped with ProLong Gold anti-fade reagent (P36930; Molecular Probes, Eugene, OR, USA), photographed Zeiss LSM 510 NLO confocal microscope. Contrast and brightness were optimized using Adobe Photoshop (Adobe Systems, San Jose, CA, USA).

## Results

### High K^+^ depolarization and voltage dependence of Ca^2+^ currents

To determine how high K^+^ solutions may increase membrane depolarizations to activate Ca^2+^ channels, we examined the effect of high-K^+^ solutions on the membrane potential (Fig. 1a) and the voltage dependence of Ca^2+^ currents activated by step depolarizations (Fig. 1b). Figure 1a shows the effect of 20 and 50 mM K^+^ on the membrane potential of a representative cell recorded with the perforated patch technique. On average, 20 and 50 mM K^+^ depolarized the membrane potential from − 53 ± 2 mV (*n* = 7) to − 39 ± 1 mV (*n* = 7) and from − 54 ± 2 mV (*n* = 7) to − 29 ± 1 mV (*n* = 7), respectively.

To determine the voltage dependence for Ca^2+^ channels activation, the cell was held at − 87 mV (after correction of − 7 mV junction potential) and the command voltage was then stepped from − 67 mV to + 43 mV with an increment of 10 mV. As the voltage-activated Ca^2+^ currents began to run down ~ 2 min after establishing the whole-cell recording mode, the voltage-dependent activation of the Ca^2+^ currents was obtained during this period*.* Figure 1b shows the Ca^2+^ currents recorded from a representative cell. Note that the currents have been obtained by subtraction of the remaining currents in 20 μM Cd^2+^. Figure 1c summarizes the *I-V* relations thus obtained from a total of 9 cells, with the arrows pointing to the membrane potentials at rest (downward arrow) and at more depolarized membrane potentials in response to 20 mM (slant arrow) and 50 mM (horizontal arrow) K^+^. Note the foot of the *I-V* relation between − 67 and − 47 mV, suggesting the presence of the low-voltage activated (LVA) Ca^2+^ currents.

### Nimodipine effects on high K^+^-induced Ca^**2**+^ rise in the SCN neurons

**Fig. 2:**
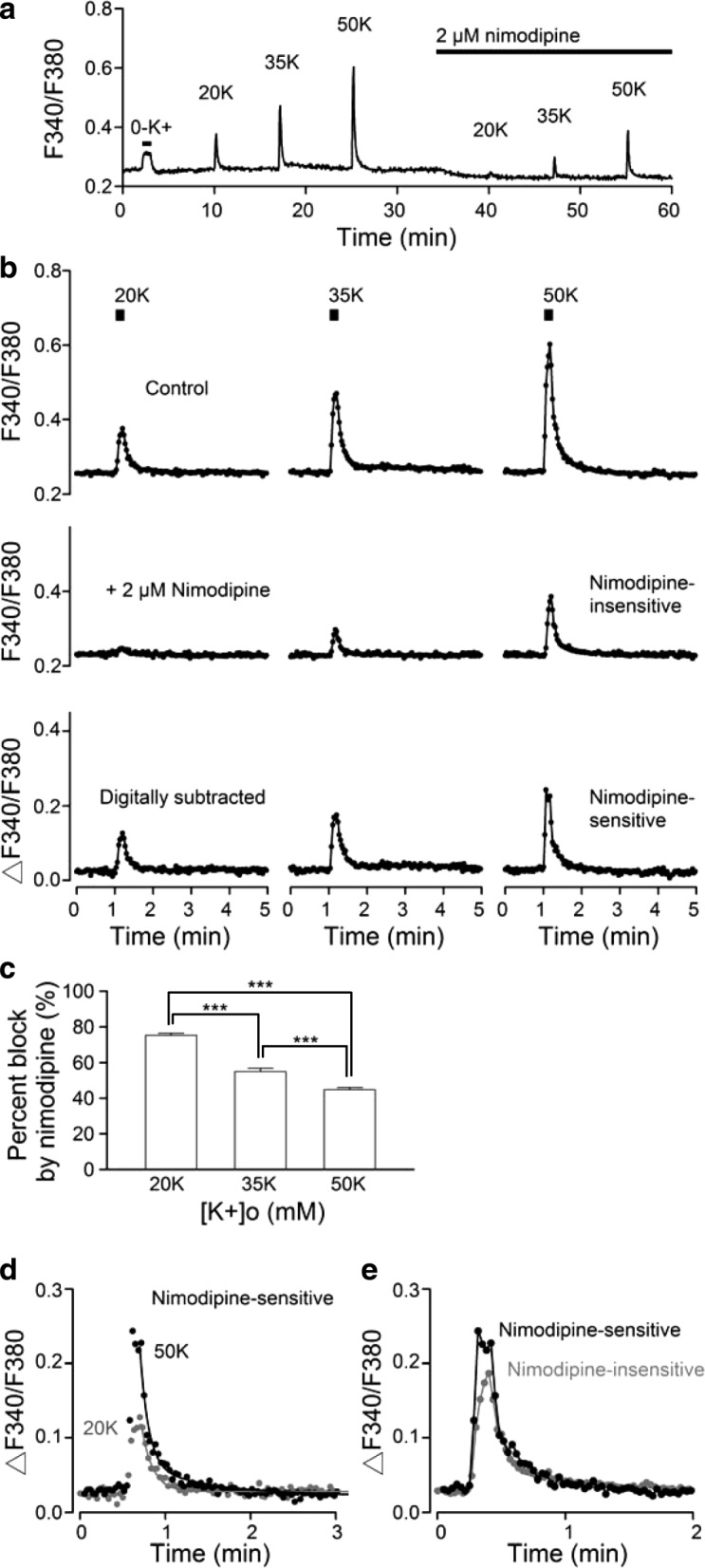
Nimodipine effects on high K^+^-induced Ca^**2**+^ transients. **a** A representative experiment to show the averaged Ca^**2**+^ response (*n* = 20 cells) to 10 s application of high K^+^ (20, 35, and 50 mM) solutions in the absence and then presence of 2 μM nimodipine. The K^+^-free (0-K^+^) solution (to block Na/K pump) was applied first to determine the condition of reduced SCN preparations and to confirm the cells being recorded are indeed neurons. **b** Averaged Ca^**2**+^ responses to 20, 35, and 50 mM K^+^ solution in control (*top* panel) and in the presence of 2 μM nimodipine (*middle* panel). *Bottom* panel shows the nimodipine-sensitive Ca^**2**+^ responses after subtraction. The peak amplitude of Ca^**2**+^ transients increased with higher K^+^ concentrations as expected. Note the much larger Ca^**2**+^ response to 20 mM K^+^ for nimodipine-sensitive (*bottom left* panel) than the nimodipine-insensitive (*middle left* panel) component. **c** Statistics showing the percent block by 2 μM nimodipine of peak Ca^**2**+^ responses to increasing concentrations of K^+^. **d** Superimposition of nimodipine-sensitive 20 K^+^- and 50 K^+^-induced Ca^**2**+^ transients for curve fitting. The grey curve for fitting the decay phase of 20 K^+^-induced Ca^**2**+^ transient was calculated with only the fast exponential decay phase, whereas the black curve for the 50 K^+^-induced Ca^**2**+^ transient was calculated with both the fast and slow exponential decay phases. **e** Comparison of nimodipine-sensitive (black) and -insensitive (grey) Ca^**2**+^ responses to 50 mM K^+^. Note the faster rate of Ca^**2**+^ rise to reach a steady state during the 10-s stimulation by 50 mM K^+^ for the nimodipine-sensitive component Ca^**2**+^ transient. Nevertheless, both components decayed in a similar way, having both a fast and slow time course, suggesting a similar mechanism for Ca^**2**+^ clearance. *** *P* < 0.001

The contribution of L-type Ca^2+^ channels to depolarization-induced Ca^2+^ rise was determined by measuring changes in [Ca^2+^]_i_ using ratiometric fluorescence recordings. All experiments began with the application of K^+^-free solution (to block Na/K pump; see ref. [37]) to determine the condition of reduced SCN preparations and to confirm the cells being recorded are indeed neurons (Fig. 2a) (see also Fig. 1 of ref. [21]). For the experiment, high-K^+^ (20, 35, and 50 mM) solutions were applied for 10 or 20 s to produce depolarization-induced Ca^2+^ transients in the absence and then presence of 2 μM nimodipine (Fig. 2a). As expected, the peak amplitude of averaged Ca^2+^ transients (*n* = 20 cells) increased with higher concentrations of K^+^ to drive membrane potential to more depolarized levels (Fig. 1). The addition of nimodipine reduced both the basal [Ca^2+^]_i_ and high K^+^-induced Ca^2+^ transients. Notably, nimodipine-induced inhibition of Ca^2+^ transients varied dependent on K^+^ concentrations (Fig. 2b, top and middle panels). Bottom panels show the digitally subtracted traces, representing the nimodipine-sensitive Ca^2+^ transients. On average, Ca^2+^ responses to 20, 35, and 50 mM K^+^ were reduced by 2 μM nimodipine by 75 ± 1% (*n* = 263), 55 ± 2% (*n* = 263), and 45 ± 1% (*n* = 263), respectively (Fig. 2c).

In addition to an increase in the peak amplitude of Ca^2+^ transients, the Ca^2+^ decay time courses also became more prolonged with increasing K^+^ concentrations. To illustrate this point, we replot the nimodipine-sensitive 20 K^+^- and 50 K^+^-induced Ca^2+^ transients for curve fitting in Fig. 2d. The theoretic curve (grey) for the decay phase of 20 K^+^-induced Ca^2+^ transient was calculated with a single time constant of 7.8 s. In contrast, the curve (black) for the 50 K^+^-induced Ca^2+^ transient was calculated with two exponential decay phases, with a fast time constant of 6 s (80% amplitude) and a slow time constant of 51 s (20% amplitude), in general agreement with our previous observation of fast and slow [Ca^2+^]_i_ decay for 50 K^+^-induced Ca^2+^ transients [21].

Interestingly, comparison of Ca^2+^ responses to 50 mM K^+^ reveals a difference in the rate of Ca^2+^ rise between the nimodipine-sensitive and -insensitive components (Fig. 2e). The nimodipine-sensitive component had a faster rate of Ca^2+^ rise to reach a steady state, contrary to the slow rise of nimodipine-insensitive Ca^2+^ transient, which did not reach a steady state during the 10 s stimulation. Nevertheless, the rate of Ca^2+^ decay appeared to be similar for both nimodipine-sensitive and -insensitive components, having both a fast and slow time course. As the NCX (NCX1) mediates the fast decay of 50 K^+^-induced Ca^2+^ transients in the rat SCN neurons [21], the result suggests a functional interaction of NCX1 with both nimodipine-sensitive and -insensitive Ca^2+^ channels to allow for NCX-mediated rapid Ca^2+^ clearance.

### CaV1.2 and CaV1.3 immunoreactivity and colocalization with NCX1

**Fig. 3:**
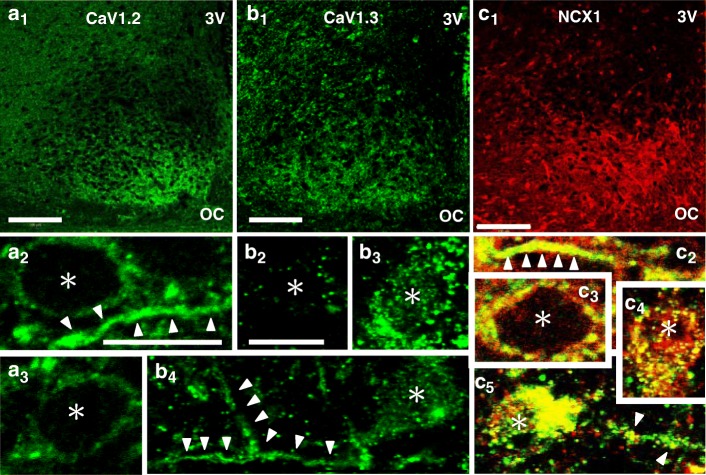
CaV1.2 and CaV1.3 immunoreactivity and colocalization with NCX1. Distribution of immunoreactivity for CaV1.2 (**a**_**1**_), CaV1.3 (**b**_**1**_), and NCX1 (**c**_**1**_) in the medial SCN. Scale bars: 100 μm. OC: optic chiasm. 3 V: third ventricle. The CaV1.2 immunoreactivity is present at or around the membrane (**a**_**2**_, **a**_**3**_) and in the process (**a**_**2**_, marked by arrowheads), but not in the cytoplasm. Intense colocalization of CaV1.2 and NCX1 can be found at the cell membrane (**c**_**3**)_ and in the process (**c**_**2**_). The scale bar is 10 μm in **a**_**2**_ and is the same in **a**_**3**_, **c**_**2**_, and **c**_**3**_. The CaV1.3 immunoreactivity also appears at or around the plasma membrane (**b**_**2**_**–b**_**4**_) and in the cell process (**b**_**4**_, marked by arrowheads), and is particularly intense in the cytoplasm of cells located in the ventral region of the SCN (**b**_**3**_, **b**_**4**_). Colocalization of CaV1.3 and NCX1 can be found in the cytoplasm of cells in the ventral region of the SCN (**c4, c5**) and in the cell process as discrete aggregates (**c**_**5**_, marked by arrowheads). The scale bar is 10 μm in **b**_**2**_ and is the same in **b**_**3**_, **b**_**4**_**, c**_**4**_, and **c**_**5**_. Asterisks mark Hoechst-stained nuclei

Nimodipine blocks the neuronal L-type Ca^2+^ channels, CaV1.2 and CaV1.3, with IC_50_ values of 0.14 and 2.7 μM, respectively [38] and at a concentration of 2 μM should block CaV1.2 and affect less than 50% of CaV1.3. Here we used CaV1.2- and CaV1.3-specific antibody to study the distribution pattern of CaV1.2 and CaV1.3 and their colocalization with NCX1 in the rat SCN (Fig. 3). Figure 3a1, b1, and c1 show the immunoreactivity (ir) for CaV1.2, CaV1.3, and NCX1, respectively, in the medial section of the SCN. High magnification images revealed intense staining of CaV1.2-ir at or around the plasma membrane (Fig. 3a2, a3) and in the cell process (marked by arrowheads; Fig. 3a2). Note the punctate double staining of CaV1.2-ir with NCX1-ir at the plasma membrane (Fig. 3c3) and along the length of a cell process (marked by arrowheads; Fig. 3c2). The CaV1.3-ir also appeared at or around the plasma membrane (Fig. 3b2–4) and in the cell process (marked by arrowheads; Fig. 3b4), and is most intense in the cytoplasm of cells located in the ventral region of the SCN (Fig. 3b3, b4). It is also the same area where the intense colocalization of CaV1.3-ir and NCX1-ir can be found (Fig. 3c4, c5). Note also the discrete aggregates of double stains of CaV1.3-ir and NCX1-ir in the cell process (marked by arrowheads; Fig. 3c5).

### Concentration-dependent effects of nimodipine on [Ca^**2**+^]_i_ and spontaneous firing

**Fig. 4:**
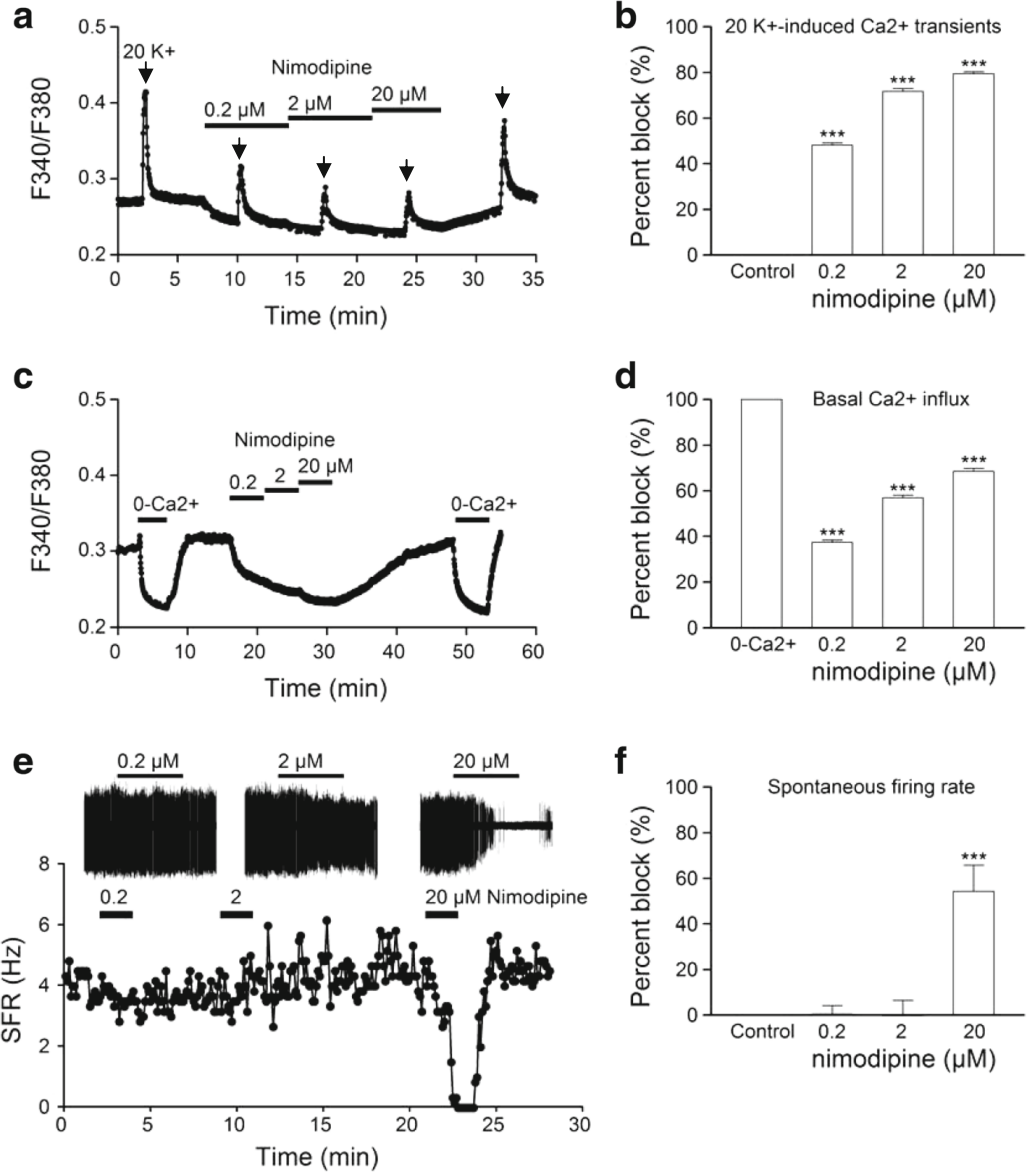
Concentration-dependent effects of nimodipine on 20 K^+^-induced Ca^**2**+^ transients (**a, b**), basal [Ca^**2**+^]_i_ (**c, d**), and spontaneous firing (**e, f**). **a** A representative experiment showing the averaged Ca^**2**+^ response (*n* = 20 cells) to 20 mM K^+^ for 20 s in control and in increasing concentrations of nimodipine. **b** Statistics showing the concentration-dependent percent block by nimodipine of 20 K^+^-induced Ca^**2**+^ responses (*n* = 260 cells). **c** A representative experiment showing the averaged Ca^**2**+^ response (*n* = 20 cells) to Ca^**2**+^-free solution and increasing concentrations of nimodipine. **d** Statistics showing the concentration-dependent percent block by nimodipine of basal Ca^**2**+^ influx as determined by Ca^**2**+^-free (0-Ca^**2**+^) solution (*n* = 344 cells). **e** The time course of change in spontaneous firing rate (SFR) of a representative cell in response to increasing concentrations of nimodipine. Insets show the cell-attached recording of firing responses to 0.2, 2, and 20 μM nimodipine. Note that 20 μM, but not 0.2 or 2 μM, nimodipine inhibited the spontaneous firing. **f** Statistics showing the concentration-dependent percent block by nimodipine of spontaneous firing (*n* = 20 cells). *** *P* < 0.001

To determine the contribution of CaV1.2 and CaV1.3, we investigated the effect of 0.2, 2, and 20 μM nimodipine on 20 mM K^+^-induced Ca^2+^ transients that were mostly mediated by L-type Ca^2+^ channels (Fig. 2). Figure 4a shows such a representative experiment (averaged response from 20 cells). The result indicates a concentration-dependent lowering of basal [Ca^2+^]_i_ and reduction of 20 K^+^-induced Ca^2+^ transients by nimodipine, with both being affected greatest by 0.2 μM nimodipine. On average, nimodipine at 0.2, 2, and 20 μM blocked the amplitude of 20 K^+^-induced Ca^2+^ transients by 48 ± 1% (*n* = 260), 72 ± 1% (*n* = 260), and 79 ± 1% (*n* = 260), respectively (Fig. 4b). After normalizing with respect to 20 μM nimodipine, the extents of inhibition by 0.2, 2, and 20 μM nimodipine became 60 ± 1% (*n* = 260), 91 ± 1% (*n* = 260), and 100 ± 0% (*n* = 260), respectively. The marked inhibition by 0.2 μM nimodipine suggests that the Ca^2+^ entering CaV1.2 channels contributes most to the nimodipine-sensitive 20 K^+^-induced Ca^2+^ rise.

To determine the contribution of nimodipine-sensitive Ca^2+^ entry to the basal Ca^2+^ influx, we compared the effects on basal [Ca^2+^]_i_ of Ca^2+^-free solution with increasing concentrations of nimodipine (Fig. 4c, d). A representative result (averaged response from 20 cells) thus obtained is shown in Fig. 4c. The lowering of basal [Ca^2+^]_i_ by the removal of external Ca^2+^ was taken as a measure of the basal Ca^2+^ influx. After normalization relative to the Ca^2+^-free solution, nimodipine at 0.2, 2, and 20 μM reduced the basal Ca^2+^ influx by 38 ± 1% (*n* = 344), 57 ± 1% (*n* = 344), and 69 ± 1% (*n* = 344), respectively (Fig. 4d). The extents of inhibition by 0.2, 2, and 20 μM nimodipine became 55 ± 1% (*n* = 344), 83 ± 1% (*n* = 344), and 100 ± 0% (*n* = 344), respectively, after normalizing with respect to 20 μM nimodipine. The result again suggests a major contribution of CaV1.2 to the nimodipine-sensitive basal Ca^2+^ influx.

The concentration-dependent effect of nimodipine on spontaneous firing was investigated using cell-attached recordings (Fig. 4e, f). Figure 4e shows a typical result obtained from a representative SCN neuron. The result indicates an inhibitory effect of 20 μM, but not 0.2 or 2 μM, nimodipine on spontaneous firing. On average, nimodipine at 0.2, 2, and 20 μM reduced the spontaneous firing rate by 0 ± 4% (*n* = 20), 0 ± 7% (*n* = 20), and 54 ± 12% (*n* = 20), respectively (Fig. 4f). Note the reduced peak-to-peak amplitude of the action currents by 2 and 20 μM nimodipine, suggesting the involvement of L-type channels in mediating the afterhyperpolarization [25].

Taken together, our results indicate that nimodipine at 0.2 and 2 μM markedly reduced basal and 20 K^+^-induced Ca^2+^ influx but had insignificant effect on spontaneous firing, suggesting an important role of CaV1.2 in the regulation of transmembrane Ca^2+^ influx but not of spontaneous firing rate. In contrast, increasing nimodipine concentrations from 2 to 20 μM markedly inhibited spontaneous firing, suggesting a possible contribution of CaV1.3 to spontaneous firing **(**but see Discussion**)**.

### Contribution of action potential-evoked Ca^2+^ influx to basal Ca^2+^ influx

**Fig. 5:**
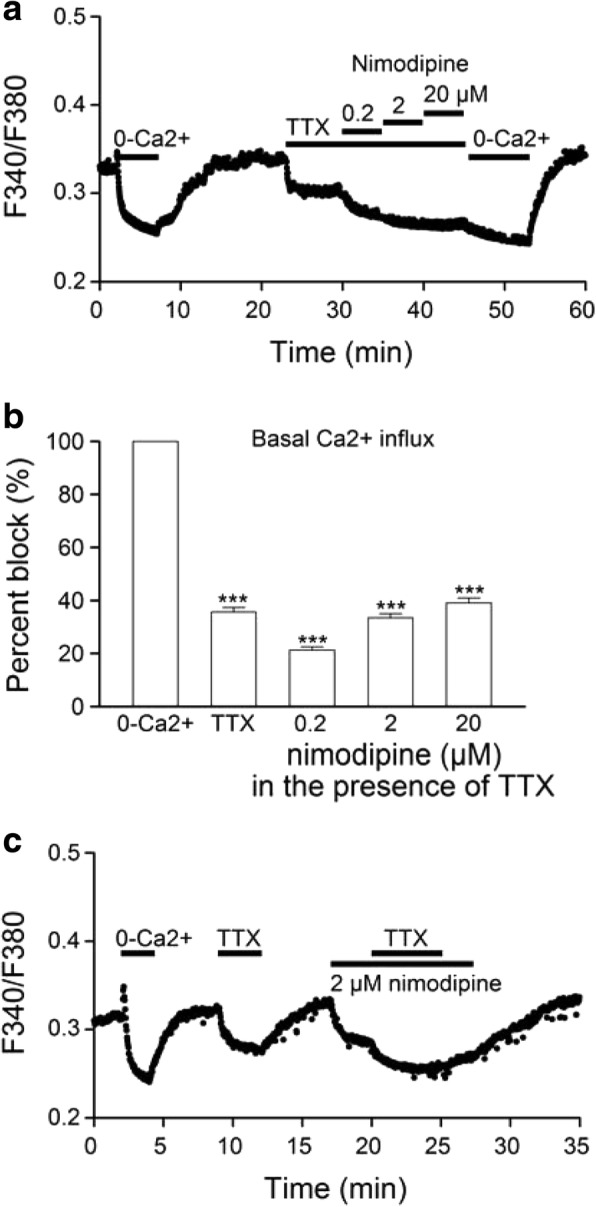
Action potential-evoked Ca^**2**+^ influx. **a** A representative experiment showing the averaged Ca^**2**+^ response (*n* = 9 cells) to Ca^**2**+^-free solution, 0.3 μM TTX, and increasing concentrations of nimodipine in the presence of TTX. **b** Statistics showing the percent block by TTX and increasing concentrations of nimodipine in TTX of basal Ca^**2**+^ influx as determined by Ca^**2**+^-free (0-Ca^**2**+^) solution (*n* = 121 cells). **c** A representative experiment showing the averaged Ca^**2**+^ response (*n* = 8 cells) to 0.3 μM TTX in the absence and then presence of 2 μM nimodipine. *** *P* < 0.001

To determine the contribution of action potential-evoked Ca^2+^ entry and the involvement of L-type channels, we investigated the effects on the basal [Ca^2+^]_i_ of 0.3 μM TTX and then increasing concentrations of nimodipine in the presence of TTX (Fig. 5). A representative result (averaged response from 9 cells) thus obtained is shown in Fig. 5a. After normalization relative to the Ca^2+^-free solution, TTX reduced the basal Ca^2+^ influx by 36 ± 2% (*n* = 121) (Fig. 5b), suggesting that TTX-sensitive action potential-evoked Ca^2+^ influx contributes to approximately one-third of the basal Ca^2+^ influx.

In the presence of TTX, nimodipine at 0.2, 2, and 20 μM further reduced basal Ca^2+^ influx by 21 ± 1% (*n* = 121), 33 ± 2% (*n* = 121), and 39 ± 2% (*n* = 121), respectively (Fig. 5b). These numbers are slightly larger than half (~ 55%) that of 38, 57, and 69% inhibitions by nimodipine in the absence of TTX (Fig. 4d), suggesting that ~ 45% of nimodipine-sensitive basal Ca^2+^ influx are mediated by the TTX-sensitive action potentials. In other words, nimodipine-sensitive basal Ca^2+^ influx comprises both TTX-sensitive and TTX-insensitive components.

Taken together the results suggest that TTX-sensitive basal Ca^2+^ influx is in part mediated by the nimodipine-sensitive L-type channels. Indeed, the magnitude of the TTX-sensitive basal [Ca^2+^]_i_ was reduced in the presence of 2 μM nimodipine as exemplified by a representative experiment shown in Fig. 5c (averaged response from 8 cells). An average block by nimodipine of 49 ± 4% (*n* = 125) suggests that TTX-sensitive basal Ca^2+^ influx comprised approximately equal shares of nimodipine-sensitive and -insensitive components. Interestingly, in the presence of both TTX and nimodipine (Fig. 5a), there remains a residual Ca^2+^ influx (25 ± 1%; *n* = 121) compared to the Ca^2+^-free solution, suggesting the presence of TTX/nimodipine-insensitive basal Ca^2+^ influx.

### The mitochondrial uncoupler preferentially increases nimodipine-insensitive Ca^**2**+^ rise

**Fig. 6:**
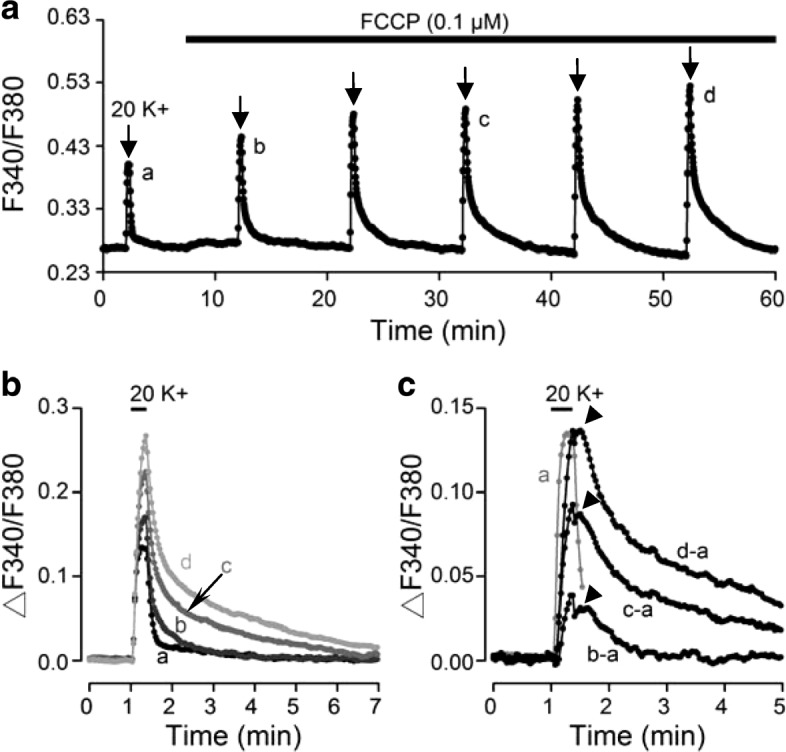
FCCP effects on 20 K^+^-induced Ca^**2**+^ transients. **a** A representative experiment showing the averaged Ca^**2**+^ response (*n* = 11 cells) to 20 mM K^+^ for 20 s in control and in the presence of 0.1 μM FCCP. **b** Superimposition of Ca^**2**+^ transients evoked in control (trace *a*) and in the presence of FCCP for 5 (trace *b*), 25 (trace *c*), and 45 min (trace *d*). Note the larger amplitude and slower decay time course for Ca^**2**+^ transients in FCCP. **c** Superimposition of subtracted traces (*b* – *a*, *c* – *a*, and *d* – *a*) to indicate the FCCP-enhanced Ca^**2**+^ transients as time progressed. The control trace (*a*) was also superimposed for better comparison of their kinetics but truncated for better visualization of the plateau-like delay prior to the decay of the FCCP-enhanced Ca^**2**+^ transients

We previously used the mitochondrial uncoupler FCCP (0.1 μM) to suggest that mitochondria regulate basal [Ca^2+^]_i_ and slow decay of 50 K^+^-induced Ca^2+^ transients [21]. As Ca^2+^ entry via CaV1.2 contributed most to both the basal and 20 K^+^-induced Ca^2+^ influx, we investigated whether mitochondria could buffer CaV1.2-mediated Ca^2+^ influx. We thus determined the effect of FCCP on 20 K^+^-induced Ca^2+^ increase (Fig. 6). For the experiment, 20 mM K^+^ solution was applied to elicit Ca^2+^ responses in the absence and then presence of 0.1 μM FCCP to reduce mitochondrial uptake as shown in Fig. 6a (average response from 11 cells). The result indicates that, in the presence of 0.1 μM FCCP, 20 mM K^+^ elicited Ca^2+^ transients with progressively larger amplitude, slower rate of Ca^2+^ rise, and slower rate of Ca^2+^ decay on repetitive application. This is best been by superimposing the Ca^2+^ transients evoked by 20 mM K^+^ solution before (trace *a*) and after (traces *b*, *c*, and *d*) the addition of 0.1 μM FCCP (Fig. 6b). Superimposition of digitally subtracted traces indicates that compared to the control Ca^2+^ transient (truncated grey trace *a*), the FCCP-enhanced Ca^2+^ transients (the subtracted traces *b* – *a*, *c* – *a*, and *d* – *a*) had slower rate of Ca^2+^ rise and decay (Fig. 6c). Specifically, the FCCP-enhanced Ca^2+^ transient rose slowly without reaching a steady state during the 20 s stimulation and decayed with mostly only a slow time course. Note the plateau-like delay (marked by arrowheads) prior to the decay of FCCP-enhanced Ca^2+^ transients.

**Fig. 7:**
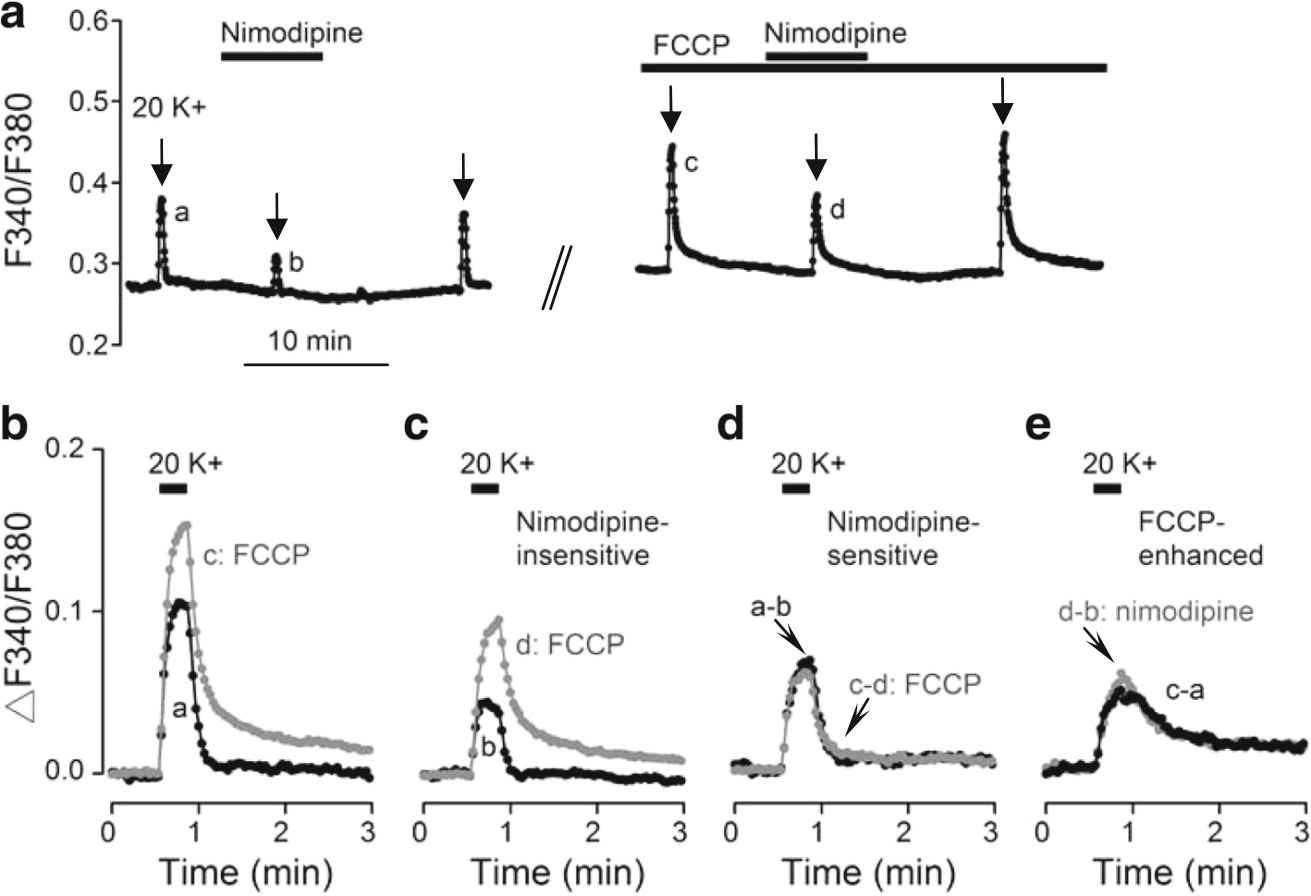
Preferential enhancement by FCCP of nimodipine-insensitive Ca^2+^ transients. **a** A representative experiment showing the effect of 2 μM nimodipine on 20 K^+^-induced Ca^2+^ response (an average of 12 cells) in the absence and then presence of 0.1 μM FCCP. Superimposition of Ca^2+^ transients to show the similar effect of FCCP on the Ca^2+^ transient evoked in the absence (**b**) and presence (**c**) of nimodipine. **d** Superimposition of subtracted traces (*a* – *b* and *c* – *d*) to show the minimal effect of FCCP on the nimodipine-sensitive Ca^2+^ transient. **e** Superimposition of subtracted traces to show that the FCCP-enhanced Ca^2+^ transients were similar in the absence (*c* – *a*) and presence (*d* – *b*) of nimodipine, indicating that FCCP selectively enhanced nimodipine-insensitive Ca^2+^ rise

As the nimodipine-insensitive Ca^2+^ transient has a slower rate of Ca^2+^ rise compared to the nimodipine-sensitive Ca^2+^ transient (Fig. 2e), the result suggests that FCCP may preferentially enhance the nimodipine-insensitive Ca^2+^ rise. To test this idea, we determined the effect of nimodipine on the 20 K^+^-induced Ca^2+^ transient before and after ~ 20–30 min into the application of FCCP (Fig. 7). Figure 7a shows the result of one such experiment (average response from 12 cells). Figures 7b, c, and d compare the effects of FCCP on total, nimodipine-insensitive, and nimodipine-sensitive Ca^2+^ transients, respectively. The results indicate that FCCP similarly enhanced the total (Fig. 7b) and the nimodipine-insensitive Ca^2+^ transient (Fig. 7c), but had little effect on the nimodipine-sensitive Ca^2+^ transient (Fig. 7d), suggesting that FCCP preferentially enhanced the nimodipine-insensitive Ca^2+^ transient. Indeed, the subtracted FCCP-enhanced Ca^2+^ transients were similar irrespective of the absence (*c* – *a*) or presence (*d* – *b*) of nimodipine (Fig. 7e). Together the result supports the idea of preferential enhancement of nimodipine-insensitive Ca^2+^ rise. On average, FCCP enhanced nimodipine-insensitive Ca^2+^ transients by 144 ± 7% (*n* = 101 cells; *P* < 0.05, paired *t*-test) and insignificantly altered nimodipine-sensitive Ca^2+^ transients by 1 ± 3% (*n* = 101 cells; *P* = 0.50, paired *t*-test). Similarly, the nimodipine-induced lowering of the basal [Ca^2+^]_i_ was also insignificantly increased by FCCP by 7 ± 9% (*n* = 97 cells; *P* = 0.21, paired *t*-test). Taken together the results suggest that mitochondria preferentially buffer Ca^2+^ influx mediated via the nimodipine-insensitive Ca^2+^ channels.

**Fig. 8:**
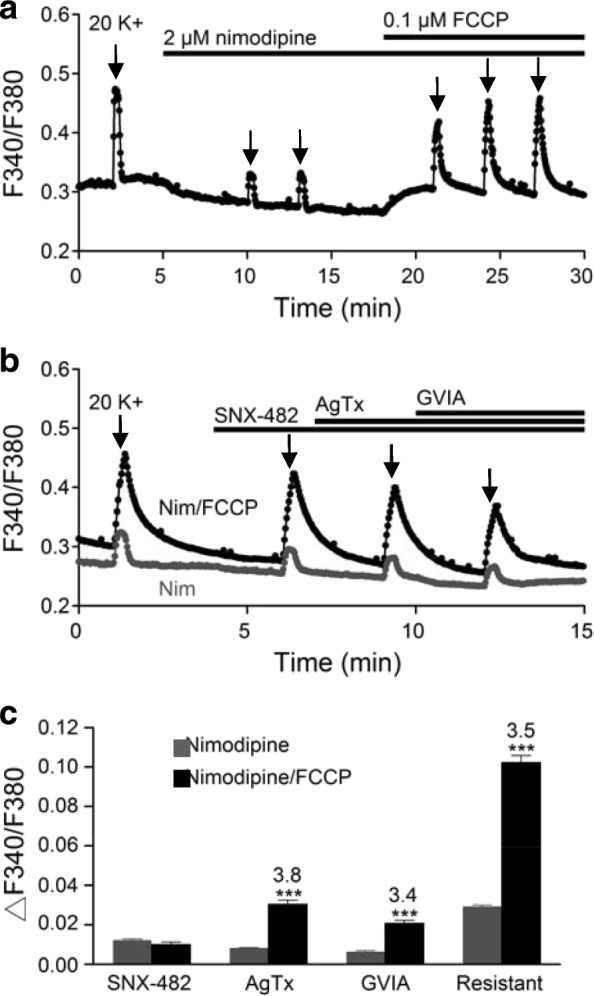
Effects of CaV2 channel blockers on nimodipine-insensitive Ca^2+^ transients. **a** A representative experiment showing the effect of FCCP on nimodipine-insensitive Ca^2+^ transients (an average of 11 cells). **b** Two representative experiments showing the effects of CaV2 channel blockers, applied additively in order of 0.2 μM SNX-482, 0.2 μM ω-agatoxin IVA, and 2 μM ω-conotoxin GVIA, on the nimodipine-insensitive Ca^2+^ transients in the absence (dark grey trace) and presence (black trace) of FCCP. **c** Summary of experiments showing the amplitude of nimodipine-insensitive Ca^2+^ transients reduced by each drug and the amplitude of Ca^2+^ transients resistant to the blocker cocktail in the absence (dark grey bars) and presence (black bars) of FCCP. Note that FCCP induced a 3- to 4-hold increase (indicated by the number on top of black bars) in the Ca^2+^ transient sensitive to ω-agatoxin IVA or ω-conotoxin GVIA or resistant to the cocktail of blockers. *** *P* < 0.001

To further determine which type of nimodipine-insensitive Ca^2+^ channels was responsible for FCCP-enhanced 20 K^+^-induced Ca^2+^ transients, we investigated the effect of CaV2 channel blockers on the Ca^2+^ transients (Fig. 8). The experiment was done in the presence of 2 μM nimodipine to block L-type Ca^2+^ channels, and then 20 mM K^+^ solution applied for 20 s to elicit Ca^2+^ transients in the absence and then in the presence of FCCP as shown in Fig. 8a. After ~ 20 min into the application of 0.1 μM FCCP, when the enhanced Ca^2+^ transients began to level off, the CaV2 channel blockers were then applied additively in order of 0.2 μM SNX-482, 0.2 μM ω-agatoxin IVA, and then 2 μM ω-conotoxin GVIA to block R-, P/Q-, and N-type Ca^2+^ channels, respectively. Figure 8b shows one such result (black trace) obtained from a representative experiment (averaged response from 17 cells). On average, SNX-482, ω-agatoxin IVA, and ω-conotoxin GVIA reduced the peak amplitude of Ca^2+^ transients by 0.0100 ± 0.0012 (*n* = 133 cells), 0.0304 ± 0.0020 (*n* = 194 cells), and 0.0208 ± 0.0015 (*n* = 187 cells), respectively, with the remaining Ca^2+^ transient amounting to a peak amplitude of 0.1023 ± 0.0034 (*n* = 194 cells).

For comparison, a separate set of experiments was performed to determine the drug effects on the nimodipine-insensitive Ca^2+^ transients in the absence of FCCP. The result of on such experiment is superimposed in Fig. 8b (dark grey trace; averaged response from 15 cells). On average, SNX-482, ω-agatoxin IVA, and ω-conotoxin GVIA reduced the peak amplitude of Ca^2+^ transients by 0.0119 ± 0.0008 (*n* = 154 cells), 0.0080 ± 0.0005 (*n* = 153 cells), and 0.0062 ± 0.0007 (*n* = 98 cells), respectively, with the remaining Ca^2+^ transient having a peak amplitude of 0.0290 ± 0.0011 (*n* = 156 cells). Note that the addition of CaV2 channel blockers may increase more than 10% of the peak amplitude of Ca^2+^ transients in some cells and these data were not included for analysis.

Figure 8c summarises the results by comparing the magnitude of the Ca^2+^ transient that was blocked by each drug and resistant to the cocktail of blockers in the absence (dark grey bars) and presence (black bars) of FCCP. The results indicate that FCCP enhances Ca^2+^ transients sensitive to ω-agatoxin IVA (*P* < 0.001; Student’s *t*-test) and ω-conotoxin GVIA (*P* < 0.001; Student’s *t*-test), but not SNX-482 (*P* = 0.18; Student’s *t*-test), suggesting that mitochondria buffer Ca^2+^ entering via P/Q- and N-, but not R-, type Ca^2+^ channels. FCCP also enhanced the remaining Ca^2+^ transient in the cocktail of blockers (*P* < 0.001; Student’s *t*-test), suggesting that mitochondria also buffer Ca^2+^ entering via the blocker cocktail-insensitive Ca^2+^ channels. Interestingly, the fold increase by FCCP (the number on top of each black bar) appeared to be similar for Ca^2+^ entering via ω-agatoxin IVA- and ω-conotoxin GVIA-sensitive and blocker cocktail-insensitive Ca^2+^ channels.

## Discussion

### Functional interaction of NCX1 and L-type Ca^**2**+^ channels

We show that the peak amplitude of high K^+^-induced Ca^2+^ transients increased with more depolarized potentials in response to increasing K^+^ concentrations (from 20, 35, to 50 mM), with its extent of block by 2 μM nimodipine decreasing from 75, 55, to 45%. The result indicates that 20 K^+^-induced Ca^2+^ rise is mediated mostly by the nimodipine-sensitive L-type Ca^2+^ channels. However, the inhibitory effect of nimodipine on the peak amplitude of high K^+^-induced Ca^2+^ transients became smaller with larger depolarizations. The result can not be accounted for by its action as an open channel blocker for the L-type Ca^2+^ channel, which would produce a larger inhibition with larger depolarizations. Rather the result indicates that larger depolarizations increase the contribution of nimodipine-insensitive Ca^2+^ rise, suggesting activation of the L-type Ca^2+^ channels by smaller depolarizations than the non-L-type Ca^2+^ channels. Indeed, in the rat SCN neurons, the L-type Ca^2+^ channels, albeit occupying only a small proportion of the total voltage-activated Ca^2+^ channels, are activated at lower voltages than other high-voltage-activated (HVA) Ca^2+^ channels [27, 28]. Nevertheless, the different buffering capacity for Ca^2+^ from different sources, including the preferential uptake by mitochondria of nimodipine-insensitive Ca^2+^ influx as demonstrated in this study, may also contribute to the observed nimodipine effects.

The decay phase of the nimodipine-sensitive 20 K^+^-induced Ca^2+^ transient could be approximated mostly with a single time constant of less than 10 s, consistent with our previous observation of NCX-mediated Ca^2+^ extrusion, which gives rise to the fast decay of the biphasic decay time course for 50 K^+^-induced Ca^2+^ transients [21]. The functional interplay of L-type Ca^2+^ channels with the NCX is also consistent with the double immunofluorescence for NCX1/CaV1.2 and NCX1/CaV1.3 at or around the plasma membrane and in the cell process.

In contrast to a single fast time constant for nimodipine-sensitive 20 K^+^-induced Ca^2+^ transients, an additional slow decay time constant was required to account for the biphasic decay time course of the nimodipine-sensitive 50 K^+^-induced Ca^2+^ transient (Fig. 2d). As the slow decay involves mitochondrial Ca^2+^ uptake (ref. [21] and this study), the result suggests a larger nimodipine-sensitive Ca^2+^ load evoked by 50 mM K^+^ recruits mitochondria to help clear Ca^2+^. Another explanation is that the prominent Ca^2+^ entry via nimodipine-insensitive Ca^2+^ channels evoked by 50 mM K^+^ may compete with that entering nimodipine-sensitive Ca^2+^ channels for extrusion via NCX1, thus rendering more Ca^2+^ mediated via nimodipine-sensitive Ca^2+^ channels available for mitochondrial uptake.

The nimodipine-insensitive 50 K^+^-induced Ca^2+^ transient also has a biphasic decay time course, which suggests similar clearance by the NCX and mitochondria, but the rate of Ca^2+^ rise is slower than the nimodipine-sensitive Ca^2+^ rise. Although the reason for the different rate of Ca^2+^ rise is not known at this moment, it may involve different Ca^2+^ buffers with different kinetics.

### L-type Ca^**2**+^ channels contribute differently to [Ca^**2**+^]_i_ and spontaneous firing

Our results reveal a differential contribution of L-type Ca^2+^ channels to the regulation of [Ca^2+^]_i_ and spontaneous firing. On the one hand, the result of concentration-dependent effects of nimodipine (0.2, 2, and 20 μM) on 20 K^+^-induced Ca^2+^ transients and basal Ca^2+^ influx (after normalization relative to the Ca^2+^-free solution) indicates a normalized percent block of 60 and 55% by 0.2 μM nimodipine, respectively, suggesting a major contribution of Ca^2+^ entry via CaV1.2 channels. On the other hand, the spontaneous firing rate was inhibited by nimodipine at a concentration of 20 μM, and was not altered by 0.2 or 2 μM nimodipine, suggesting an insignificant contribution of CaV1.2 to spontaneous firing. Together the results suggest that CaV1.2 mediates most of the nimodipine-sensitive Ca^2+^ rise, but has insignificant effect on spontaneous firing.

The insignificant effect of 2 μM nimodipine on spontaneous firing presented in this study is consistent with the result obtained from also rat SCN neurons in dissociation [27] but at odds with that obtained from rat SCN neurons in slices [28]. Furthermore, 10 μM nimodipine also does not significantly alter spontaneous firing in rat SCN neurons in slices [25]. The reason for the discrepancy is not clear at this moment.

On the other hand, 20 μM nimodipine markedly inhibits spontaneous firing rate in rat SCN neurons. The result may be taken to suggest a contribution of CaV1.3 to the generation of spontaneous firing as demonstrated in the genetically altered mouse SCN neurons (10 μM nimodipine, ref. [30]). Nevertheless, although nimodipine at a concentration of 10 μM blocks CaV1.3 [38], at this concentration it is nonspecific and also blocks P/Q- and N-type HVA (see ref. [39]) and CaV3.1-type LVA Ca^2+^ channels [40]. Thus, the inhibition of firing rate by 20 μM nimodipine could be a result of combined inhibition of all these Ca^2+^ channels.

### Properties of the basal Ca^**2**+^ influx in the SCN neurons

Our results also reveal that the basal Ca^2+^ influx (after normalization relative to the Ca^2+^-free solution) during the day is mostly mediated by TTX- and nimodipine-sensitive Ca^2+^ influx (Figs. 4 and 5). Specifically, nimodipine at 0.2, 2, and 20 μM reduced basal Ca^2+^ influx by 38, 57, and 69%, respectively, and, even with TTX to block the generation of Na^+^-dependent action potentials, still reduced it by 21, 33, and 39%, respectively. The result indicates that nimodipine-sensitive basal Ca^2+^ influx comprises both TTX-sensitive and -insensitive components. The TTX-sensitive component is attributable to Na^+^ action potential-evoked nimodipine-sensitive Ca^2+^ entry (ref. [27] and this study), whereas the TTX-insensitive component may partly be attributed to Ca^2+^ spikes [26] or Ca^2+^-mediated oscillations in the membrane potential [27, 28]. Indeed, nimodipine has been shown to block the Ca^2+^-mediated oscillations in TTX in the rat SCN neurons [27, 28]. Nevertheless, the possibility exists that TTX may reduce [Na^+^]_i_ to lower the levels of [Ca^2+^]_i_ by enhancing Ca^2+^ extrusion via the plasmalemmal NCX and/or by reducing Ca^2+^ efflux to the cytosol via the mitochondrial NCX (NCLX).

On the other hand, TTX reduced the basal Ca^2+^ influx by 36%, and both nimodipine-sensitive and -insensitive components contributed approximately equally to the TTX-sensitive basal Ca^2+^ influx. In dissociated rat SCN neurons studied with the action potential clamp technique, the action potential clamp-evoked Ca^2+^ current also comprises both nimodipine-sensitive and -insensitive components [27], but the nimodipine-insensitive component is considerably larger (~ 4 times as large as the nimodipine-sensitive component as estimated from Fig. 10B of ref. [27]). Although it is not possible to directly compare our results with those of Jackson et al. [27], the preferential uptake of nimodipine-insensitive Ca^2+^ influx into mitochondria would predict a reduced contribution of nimodipine-insensitive Ca^2+^ current to action potential-evoked Ca^2+^ rise.

Importantly, our results also reveal a portion (25%) of the basal Ca^2+^ influx that is not sensitive to the combined presence of TTX and nimodipine. Possible sources for the TTX/nimodipine-insensitive basal Ca^2+^ influx include the LVA Ca^2+^ channels [26] and the voltage-independent Ca^2+^-permeable channels such as the transient receptor potential channels. Nevertheless, it cannot be excluded the possibility that NCX operating in the reverse mode [21] may also contribute to the basal Ca^2+^ influx. Further work is needed to better determine the nature of TTX/nimodipine-insensitive basal Ca^2+^ influx. It should be noted that although we only address the issue of transmembrane Ca^2+^ influx in this study, internal Ca^2+^ release also plays an important role in SCN physiology (see ref. [41]).

### Mitochondria preferentially buffers nimodipine-insensitive Ca^**2**+^ influx

One of our major finding is the preferential uptake by mitochondria of depolarization-evoked nimodipine-insensitive Ca^2+^ influx. This is demonstrated by the observation that FCCP markedly increased the amplitude and prolonged the decay kinetics of the nimodipine-insensitive Ca^2+^ transient, but had minimal effect on the nimodipine-sensitive Ca^2+^ transient. Experiments with sequential addition of CaV2 channel blockers further reveal that FCCP enhances nimodipine-insensitive Ca^2+^ rise mediated by Ca^2+^ entering N- and P/Q- but not R-type Ca^2+^ channels. FCCP also enhances Ca^2+^ rise mediated by Ca^2+^ entering the blocker cocktail-insensitive Ca^2+^ channels. While it remains to be determined the nature of Ca^2+^ channels insensitive to the cocktail of blockers for L-, N-, P/Q-, and R-type Ca^2+^ channels, our preliminary result indicated that the blocker cocktail-insensitive Ca^2+^ transients were partially blocked by 20 μM Cd^2+^ and mostly blocked by 200 μM Cd^2+^, suggesting most likely an origin of T-type Ca^2+^ channels.

On the other hand, the lowering effect of nimodipine on basal [Ca^2+^]_i_ is also not much affected by the presence of FCCP (Fig. 7), suggesting that FCCP also has a minimal effect on the nimodipine-sensitive basal Ca^2+^ influx. In other words, the FCCP-induced increase in basal [Ca^2+^]_i_ (ref. [21] and this study) should also most likely be mediated by the nimodipine-insensitive basal Ca^2+^ influx. Nevertheless, experiments with sequential addition of CaV2 channel blockers suggest that Ca^2+^ entry via N-, P/Q-, and R-type Ca^2+^ channels contributes only partially to the FCCP-induced increase in basal [Ca^2+^]_i_. It remains to be determined whether the FCCP-induced increase in basal [Ca^2+^]_i_ may also involve the blocker cocktail-insensitive Ca^2+^ channels. Further work is needed to resolve this issue.

The preferential uptake by mitochondria of Ca^2+^ entry via nimodipine-insensitive over nimodipine-sensitive Ca^2+^ channels may allow nimodipine-insensitive Ca^2+^ channels to play a role in regulating mitochondria-associated functions [42], suggesting that Ca^2+^ entry via different sources may be regulated differently to play different roles in SCN physiology. Indeed, in cultured superior cervical ganglion and hippocampal neurons, there is also a preferential uptake by mitochondria of nimodipine-insensitive CaV2-mediated Ca^2+^ entry, likely due to the close relationship between CaV2 channels and SERCA in subsurface endoplasmic reticulum, with consequential lowering of CaV2-mediated Ca^2+^ rise to limit its impact on gene expression [43].

### Functional implications

The contribution of CaV1.2 in mediating most of the basal Ca^2+^ influx accords with an important role of nimodipine-sensitive Ca^2+^ influx in regulating PER2 oscillation [19]. Since NCX1 appears to interact with CaV1.2 to regulate nimodipine-sensitive somatic Ca^2+^ rise, our results suggest a role of NCX1 in conjunction with L-type Ca^2+^ channels in the regulation of clock gene oscillation. Furthermore, the intense labelling of CaV1.2 (and CaV1.3) in the cell processes suggests that L-type channels, along with NCX1, may also regulate Ca^2+^ levels in these compartments, most likely playing a role in the intercellular communication, for example, by regulating the release of neuropeptides. In this context, it is worth mentioning that TTX- or nimodipine-dependent long-range interactions between the ventrolateral and dorsomedial SCN confers the resistance of SCN to temperature entrainment, with the inter-regional communication mediated by a signaling pathway that may involve VIP and AVP [19].

Neuropeptide signaling is critical for proper functioning of the SCN [44]. Experiments with reverse-microdialysis perfusion from the hamster SCN indicate that the resting release of AVP, VIP, and GRP depends on [Ca^2+^]_i_, being increased by high K^+^ solutions and reduced by a cocktail of Ca^2+^ channel blockers in Ca^2+^-free solution [16, 17]. Furthermore, putative peptidergic dense-core vesicles are found to be released, by high-K^+^ solution in a Ca^2+^-dependent manner, from axonal terminals and somatodendritic areas of the rat SCN neurons [45]. The contribution of both nimodipine-sensitive and -insensitive Ca^2+^ channels in mediating the basal Ca^2+^ influx and, along with NCX1, in shaping high K^+^-induced Ca^2+^ rise suggests their possible involvements in regulating the release of neuropeptides. It remains to be determined whether NCX1 indeed interact with Ca^2+^ channels to regulate neuropeptides release in the SCN. As a first step toward this end, we recently investigated the colocalization of neuropeptides with NCX2, which unlike NCX1 has a more restricted distribution to the ventral region of the SCN [21]. We employed double and triple staining immunofluorescence to demonstrate selective localization of NCX2 with VIP, GRP, and VIP/GRP, but not with AVP. Importantly, the presynaptic marker Bassoon was found to colocalize with NCX2/GRP and NCX2/VIP, suggesting a role of NCX2 in the regulation of release of VIP and GRP [46].

Last, mitochondria preferentially buffer entering Ca^2+^ via nimodipine-insensitive Ca^2+^ channels. As Ca^2+^ entering mitochondria is known to activate dehydrogenase to increase oxidative phosphorylation [42], our results suggest a possible role of nimodipine-insensitive Ca^2+^ influx in linking neuronal activity and energy metabolism. Thus, identifying the sources for nimodipine-insensitive Ca^2+^ influx may help better understand the in-phase circadian rhythms in neuronal activity, intracellular Ca^2+^ concentrations, and metabolic activity in the SCN [47]. Our results suggest that all (L-, N-, P/Q-, R-, and most likely also T-) types of Ca^2+^ channels are involved in mediating basal [Ca^2+^]_i_ and depolarization-induced Ca^2+^ influx. Interestingly, we recently found that glucose shortage compromises mitochondrial respiration to activate ATP-sensitive K^+^ channels in the AVP-SCN neurons [48]. Taken together, the results suggest that glucose shortage may deregulate Ca^2+^ homeostasis by inhibiting respiration and thus reducing mitochondrial Ca^2+^ uptake. As reduced glucose availability is known to attenuate circadian responses to light in mice [49], it would be interesting to know whether a compromised mitochondrial function might be involved in the process.

## Conclusions

While both nimodipine-sensitive CaV1.2 and CaV1.3 are expressed and colocalize with NCX1 in the rat SCN, CaV1.2 appears to mediate most of the nimodipine-sensitive Ca^2+^ rise and contributes to most of the basal Ca^2+^ influx. Furthermore, whereas NCX1 mediates rapid Ca^2+^ clearance of both nimodipine-sensitive and -insensitive Ca^2+^ transients, mitochondria preferentially buffer Ca^2+^ entry via nimodipine-insensitive N-, P/Q-, and most likely also T-type Ca^2+^ channels. The differential regulation of transmembrane Ca^2+^ influx by NCX and mitochondria suggests that Ca^2+^ entering via different Ca^2+^ sources may be regulated differently to play different roles in SCN physiology.

## Abbreviations

AVP: Arginine vasopressin
CRE: Ca^2+^/cAMP-response element
FCCP: Carbonyl cyanide-p-trifluoromethoxyphenylhydrazone
GRP: Gastrin releasing peptide
HVA: High-voltage-activated
LVA: Low-voltage activated
NCLX: Mitochondrial NCX
NCX: Na^+^/Ca^2+^ exchanger
PMCA: Plasmalemmal Ca^2+^-ATPase
SCN: Suprachiasmatic nucleus
SERCA: Sarco(endo)plasmic reticulum Ca^2+^-ATPase
VIP: Vasoactive intestinal peptide
